# The Sigma Ring and Other Distinctive Features of Surface Potentials of Group 1 Systems

**DOI:** 10.1021/acs.jpca.6c02529

**Published:** 2026-07-08

**Authors:** Kelling J. Donald, Victoria Rankin, Brice Di Carlo, Jonathan E. Findley, Ethan B. Leonard

**Affiliations:** Department of Chemistry, Gottwald Center for the Sciences, 6888 University of Richmond, Richmond, Virginia 23173, United States

## Abstract

For a sigma hole induced on a terminal atom, such as a halogen atom (X) in a molecule R–X, it is commonly expected that the maximum potential in that sigma hole will arise along the extension of the R–X bond, opposite the overlap region. We find, however, that for the majority of group 1 halides (MX) that expectation fails. Conventional sigma holes appear on Na in its halides and hydrides, but not so for M = K, Rb, or Cs. Instead, a sigma ring—a latitudinal ring maximum—is observed in the surface potentials of those molecules. That anomaly is examined, and its entailments for structure and bonding in acid–base complexes involving metal halides are found to be substantial. Li generally shows the same behavior as Na, but there are curious exceptions. Certain even more consequential features of the surface potentials themselves—beyond the presence or absence of a sigma hole or ring—including the fact that the less electronegative halides lead to the strongest surface potentials on group 1 atomic centers in MX molecules, are demonstrated and rationalized. Our observations allow us to make and evaluate predictions about metal halides and hydrides as bonding partners in noncovalent interactions.

## Graphical abstract

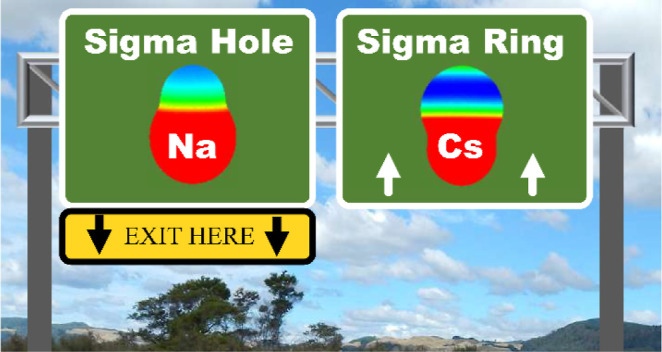

## Introduction

It is now well established that terminal halogen substituents, X, can imitate hydrogen in its role as an electron acceptor in hydrogen bonds (R–H···Base).^1–3^ For halogens, such R–X···Base interactions have come to be called, analogously, halogen bonds.^4^ The observation that anisotropy in the surface electrostatic potential, ESP, about the terminal halogens appears to drive such R–X···Base type bonding interactions led eventually to what is now well-known as the sigma hole concept.^5,6^ Those R–X···Base interactions in which the X sites behave like an electron acceptor were fascinating because halides are classically seen to be electron-rich. The halogens are some of the most electronegative atoms in the periodic table, are characteristically assigned negative net (computational) charges in their compounds,^7^ and are known to be electron donors,^8,9^ all features that do not forecast Lewis acidity.

If R is sufficiently electron-withdrawing, however, a halogen, X, site can be polarized quite significantly upon R–X bond formation. As electron density accumulates on one side of X (in the R–X overlap region), an area of electron deficiency, which is referred to nowadays as a *sigma hole*, arises on the other side of X around the extension of the bond axis—in the area indicated by the “•” in R–X•. That depletion in the electron density can be so substantial that the net electrostatic potential in that region on X becomes quite positive, allowing R–X to establish a stabilizing noncovalent interaction with a base.^7,10^

For any given sigma hole, the most positive surface ESP value (*V*_s,max_ for that sigma hole) sits inevitably at its center. In the case of a diatomic R–X molecule, for instance, *V*_s,max_ would sit exactly at the pole of X along the extension of the bond, with *V*_s_ becoming less positive (more negative) as we move away from the pole over the surface of X toward the overlap region. Since the lone pairs of a terminal halogen atom form an electron-rich belt around the middle or equator of X in the molecule (perpendicular to the R–X bond axis), the location of the sigma hole on X in an R–X molecule is predictable and well-defined. Importantly, the size of the sigma hole and the magnitude of *V*_s,max_(X), depend significantly on the size and polarizability of X and increase monotonously as X gets larger and softer going down group 17 from F to I, for a given R group.^10^

The same general mechanism—polarization due to σ bond formation leading to or strengthening regions of positive potential on an atomic center—can also lead to the formation of sigma holes on central atoms (M).^11–16^ SiF_4_, for instance, has four sigma holes on Si.^11,12,14^ In principle, the polarization of M by any substituent, R, in an MR_*x*_ molecule can lead to “*x*” sigma holes on M, one opposite each R–M bond (some of which may be hidden if, for instance, one M–R bond is trans to another), and sigma hole type R_*x*_M···Base interactions to such polarized central M atoms have been described for elements from across the periodic table.^17–20^

For group 1 atoms, where *x* = 1, such RM···Base interactions are possible as well. The analogy to hydrogen bonding is probably even more fitting in this case since—despite petitions to relocate it^21^—hydrogen is affiliated with group 1, and those elements have the same ns^1^ valence shell electron configuration as hydrogen. Moreover, since group 1 atoms are quite electropositive, the presence of a positive potential on the atomic surface in almost any molecule with terminal group 1 atoms, even if R is only mildly electron-withdrawing, is not surprising at all. Noncovalent lithium^22–27^ and sodium bonding^28,29^ —not to be confused with the (dative) covalent bonding in rhomboid (MX)_2_ clusters, for instance,^27^ have in fact been described in the literature.

We find, however, that the situation in group 1 is much more complicated than an extrapolation from hydrogen bonding. As we go down group 1 from Li to Cs, the response of the atoms to polarization changes substantially with marked consequences for complex formation. We point specifically to a sigma ring phenomenon that arises for group 1 metal centers with increasing likelihood and prominence as M gets larger. The influence of polarization on surface potentials of group 1 atoms is also found to be qualitatively very different from that observed for the halogens.

## Computational Methods

The chemical species considered in this investigation have been optimized without constraints using the Gaussian (G16) software.^30^ All of the structures reported herein have been confirmed by harmonic vibrational frequency analyses to be minima on the relevant potential energy surfaces, each exhibiting no imaginary frequency as evaluated at the same level of theory deployed for the structural optimization. For all of those G16 calculations, we employed the def2-TZVPP,^31,32^ all-electron basis sets for elements above period 5 and the associated default ECPs and valence basis sets as defined in the G16 software for Rb, Cs, and I, in combination primarily with the ωB97XD method.^33^ That method takes dispersion into account, is computationally less costly than several of the alternative methods, and is effective in predicting structural and thermodynamic properties of small molecules or weakly bound complexes such as those considered in this work.^33^ The MP2­(full)^34–36^ and CCSD­(full)^37,38^ methods were employed as well to establish at key junctures the method (in)­dependence of certain observations.

To assess whether certain unusual features observed in the surface electrostatic potentials of group 1 halide and other molecules are actual physical phenomena and not anomalies associated with the use or choice of ECPs, we employed four other classes of basis sets: the aug-cc-pVTZ^39,40^ basis sets for all elements above period 5 except K and the Stuttgart–Cologne small-core Dirac–Fock effective core potentials (ECPs) and valence basis sets^41^ for K, Rb, Cs,^42^ and I,^43^ and the all-electron dyall-acv3z,^44,45^ x2c-TZVPPall,^46^ and ANO-RCC-VTZP^47,48^ basis sets.^49^ Interaction energies are computed for selected sigma hole type complexes, with basis set superposition error (BSSE) estimated using the counterpoise correction^50,51^ as implemented in the G16 suite of programs.

All ESP maps shown in this work were generated using the GaussView 6 program,^52^ and simple ball and stick molecular structures were produced using the Chemcraft graphical user interface.^53^ The Multiwfn software^54,55^ has been used to determine the magnitude and positions of ESP extrema on molecular surfaces, and the natural bond orbital analysis tool implemented in the G16 software was used to compute the point charges reported in this work.

## Results and Discussion

Two sets of computed surface electrostatic potentials for the group 1 fluorides, MF, for M = Li to M = Cs are shown in Figure 1. They were obtained using the def2-TZVPP basis sets (relying on ECPs for Rb, Cs, and I), and the all-electron dyall-acv3z basis sets. A comparison of the results from those two sets of calculations allows us to assess the reliability of the ECPs in identifying unusual properties in the surface potentials of compounds of heavy group 1 atoms, or conversely to demonstrate that the phenomena under discussion here are not artifacts of ECPs. The corresponding results for three other types of basis sets are provided in Figure S1 in the Supporting Information.

**Figure 1:**
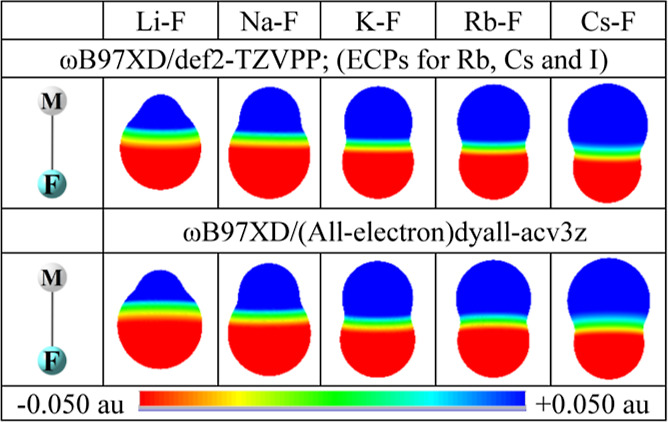
Computed ESP maps (on the 0.001 au iso-surface) for group 1 fluorides, all on the ESP color scale indicated. For each map, the MX molecule is oriented with M at the top as shown in the left column. Note: for electric potential 1 au = *E*_h_·e^–1^; for charge density 1 au = 1 e·a_o_^–3^.

For each molecule, the ESP maps in Figure 1 are similar at both levels of theory, and qualitatively identical results are obtained with the three other (including two all-electron) basis sets considered (see Figure S1). The F site (lower region in all of the ESP plots shown in Figure 1) has, as expected, very negative surface potentials, with an extremum at the pole of F opposite the M–F bond. The surface potentials for the M sites are generally positive indeed (blue in Figure 1), but there is much more to see than those ESP maps reveal.

The common scale used in Figure 1 is helpful; it allows us to see definitively the general partition between the positive (greenish blue to blue) and negative (yellow to red) sections of the ESP maps. If the (±0.05 au) color scale used for the ESP maps in Figure 1 is decompressed, however, and molecule-specific scales are used, variations in the ESP values (*V*_s_) become clearer, and an intriguing detail—the sigma ring—is exposed (Figure 2). We find that the maximum in the ESPs about the M centers (*V*_s,max_(M)) occurs at the pole on M only in certain cases—i.e., for M = Li (in most cases), and Na. For M = K, Rb, and Cs, the potential at the pole (*V*_s_(pole)) is in fact a local *miniumum*! That is, although *V*_s_(pole) is positive, the ESP increases somewhat as we move away from the pole toward the equator of the M basin, and achieves its maximum as a latitudinal ring at some point between the pole and the equator on the M surface.

**Figure 2:**
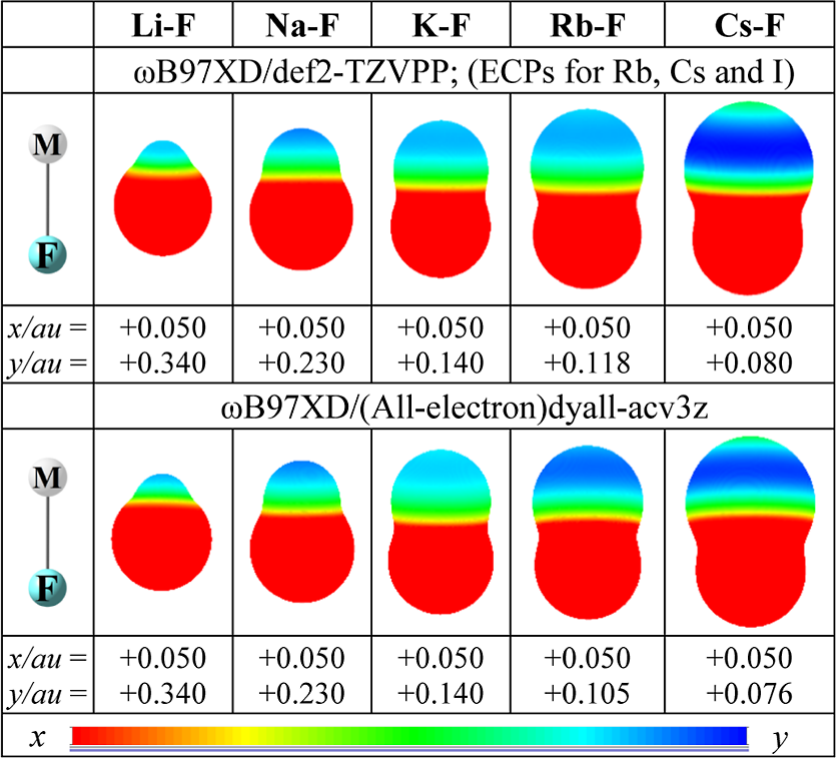
Computed ESP maps (on the 0.001 au iso-surface) for MF molecules, each with *x*/*y* ranges selected to make the positive extrema on M as prominent as possible. Slightly different “*y*” values were used for both the RbF and CsF cases to improve contrast for a given level of theory.

### The Reality of an Anomaly

Figure 2 maps the same surface potentials from Figure 1, but with individual color scales for each molecule. We use the same scale across five different levels of theory (three shown only in Figure S2) for LiF, NaF, and KF, but deviate from that ideal a bit for RbF and CsF (see Figure 2) to ensure as strong a contrast as we could manage over the ESP surface around M.

For M = K, Rb, and Cs, a close look at the ESP maps shows, with increasing ease as M gets larger, that the most positive region on the M surface (top section of each map in Figure 2) is not at the pole of M along the extension of the M–F bond where *V*_s,max_ is traditionally expected to appear. Instead, the most positive potential(s) appear as a ring around a latitudinal region—a spherical segment or frustum—of the M site, most obviously in Figure 2 around Cs in CsF.

To put our discussion on a more quantitative footing, we present in Table 1 the computed extrema in the electrostatic potentials about the M center in the group 1 fluorides. Remarkably, Na exhibits no sigma ring, even though a very small ring is observed for M = Li with two of the model chemistries considered (see Table 1 and the Supporting Information). That ring is so tight around the pole of the small Li site in F–Li, however, and the difference between *V*_s_(pole) and *V*_s_(ring) (defined here as Δ*V*_(r–p)_ = *V*_s_(ring) – *V*_s_(pole)) is so small that it is imperceptible in Figure 2.

**Table 1:** Extrema in the Computed Electrostatic Potentials (in kcal·mol^–1^ units)^a^ Obtained at the Pole (*V*_s_(pole)) and Ring (*V*_s_(ring)) on Group 1 M Atoms in MF Molecules Using the ωB97XD Method and the Basis Sets Indicated^b^

M	Li	Na	K	Rb	Cs
def2-TZVPP or ECP + Valence Basis Set					
*V*_s_(pole)	176.0	131.1	76.2	65.8	42.6
*V*_s_(ring)	**176.8**	-	**77.7**	**67.9**	**50.1**
Δ*V*_(r–p)_	0.8	-	1.5	2.1	7.5
(All-electron) dyall-acv3z					
*V*_s_(pole)	**182.3**	**130.8**	74.6	59.2	40.3
*V*_s_(ring)	-	-	**76.2**	**62.5**	**46.7**
Δ*V*_(r–p)_	-	-	1.6	3.3	6.4

a Conversion factors: 1 kcal·mol^–1^ = 4.336411 × 10^–2^ eV = 1.593601 × 10^–3^ au.

b The difference Δ*V*_(r–p)_ = *V*_s_(ring) – *V*_s_(pole) is given where relevant. Note that, when the sigma ring is present, the less positive potential at the pole on M, *V*_s_(pole), is a local minimum.

Where a sigma ring exists, *V*_s_ decreases monotonously toward the pole and toward the bonding region, so *V*_s_(ring) > *V*_s_(pole) such that Δ*V*_(r–p)_ is positive and *V*_s_(ring) ≡ *V*_s,max_ on the surface of the MF molecule. For each molecule in Table 1, the global *V*_s,max_ values (i.e., *V*_s_(pole), or *V*_s_(ring) where it exists) is in bold type. The data in Table 1 confirm, in line with the evidence in Figure 2, that Δ*V*_(r–p)_ increases rapidly going from K to Cs, (jumping by 6.0 and 4.8 kcal·mol^–1^ for the two levels of theory shown in Table 1, for example) and that this sigma ring phenomenon is most strongly exhibited in the Cs system (see also Tables S1–S5 and Figure S2 in the Supporting Information).

Of note, however, the actual magnitude of *V*_s,max_ decreases monotonously going from Li to Cs in Table 1, even as the ring phenomenon emerges and Δ*V*_(r–p)_ increases. Indeed, all of the levels of theory agree on a rapid weakening in the maximum surface potential on M of over 125 kcal·mol^–1^ going from Li to Cs. That variation is not discernible in Figure 1 or 2. We can compare all of the maps in Figure 1 with each other, but that color scale (even as it helps to establish clearly the positive and negative regions of the molecules) is too compressed for us to see the gradients in the ESPs across the molecular surfaces. In Figure 2, the carefully tuned scales are molecule-specific, emphasizing the presence and positions of the sigma holes and rings, but those maps cannot be compared across columns since the scales are different. So, Table 1 goes a long way to avert misperceptions, and even though the wide range in the ESP values on the surfaces of these MF systems makes it difficult to find a common and effective scale for all purposes, we will make another attempt in the next section.

Before taking that next step, however, we should point out that the ring phenomenon is not unique to the ωB97XD density functional method. Qualitatively identical profiles are observed in the electrostatic potentials of the group 1 fluorides using both the MP2­(full) and CCSD­(full) methods (Figure 3) in combination with the def2-TZVPP class of basis sets. As in Figure 2, we present in Figure 3 surface potential maps that have been plotted in ranges that allow for the exhibition of the cap or band of the most positive potentials on the surface of each M fragment in group 1 MF molecules.

**Figure 3:**
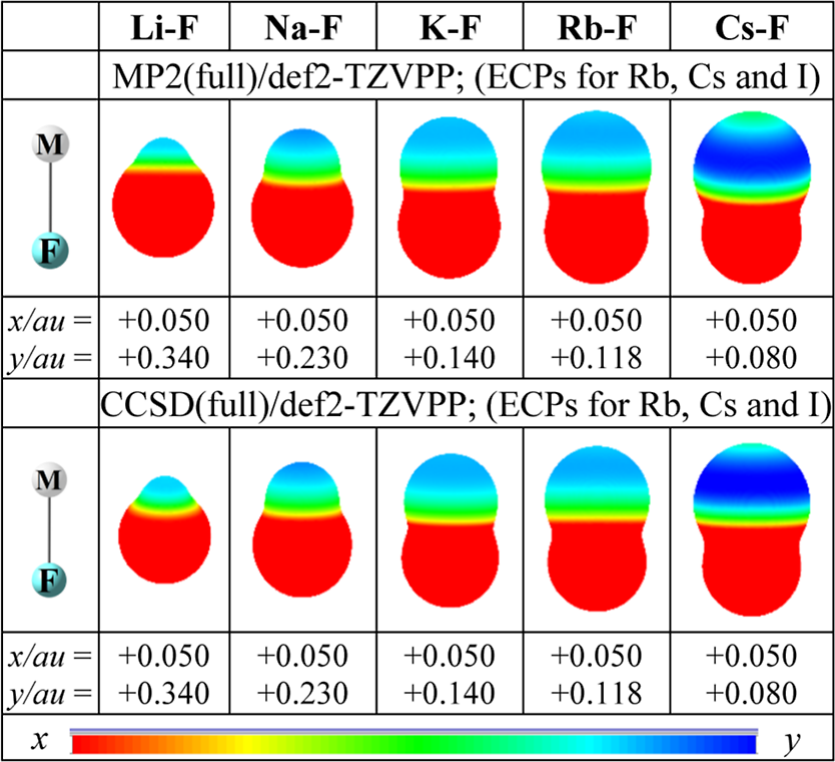
Computed MP2­(full) and CCSD­(full) surface ESP maps (on the 0.001 au iso-surface), with *x*/*y* ranges selected to make the positive extrema on M as prominent as possible.

Here again (see Figure 3) the rings become more prominent as we go from KF to CsF, not because *V_s_* (ring) increases, but because - even as *V_s_* (ring) decreases - the *difference* between V_s_(ring) and V_s_(pole) increases going from M = K to M = Cs: so an increasingly sharp visual contrast is achievable as the color scale of the ESP map is tuned for the best possible illustration of the presence of a ring.

The actual values of the MP2­(full) and CCSD­(full) ESP extrema (*V*_s_(pole) and *V*_s_(ring)) on the M surfaces of the MF molecules are listed in Table 2. The data from both of those post-HF ab initio methods are in good qualitative agreement with each other and with the density functional (ωB97XD) results in Table 1, confirming, for instance, the substantial increase in Δ*V*_(r–p)_ going from KF to CsF, and predicting the presence of a small ring on Li in LiF but a simple sigma hole on NaF.

**Table 2:** Extrema in the Computed Electrostatic Potentials (in kcal·mol^–1^ units) Obtained at the Pole (*V*_s_(pole)) and Ring (*V*_s_(ring)) on Group 1 M Atoms in MF Molecules Using the MP2­(full) and CCSD­(full) Methods and the def2-TZVPP Type Basis Set^a^

M	Li	Na	K	Rb	Cs
MP2(Full)					
*V*_s_(pole)	*175.0*	**129.0**	*76.9*	*65.1*	*43.0*
*V*_s_(ring)	**176.0**	-	**77.6**	**66.8**	**49.9**
Δ*V*_(r–p)_	1.0	-	0.7	1.7	6.9
CCSD(Full)					
*V*_s_(pole)	*175.0*	**129.1**	*77.5*	*65.2*	*44.1*
*V*_s_(ring)	**175.8**	-	**78.1**	**66.8**	**50.6**
Δ*V*_(r–p)_	0.8	-	0.6	1.6	6.5

a The Δ*V*_(r–p)_ values are provided as well.

Beyond the choice of method, we can confirm as well that the sigma ring phenomenon is not an accident of the choice of electron isodensity surface (0.001 au). Figure 4 shows the computed ωB97XD, MP2­(full), and CCSD­(full) electrostatic potential maps for Cs for five different iso-surfaces: 0.01, 0.005, 0.001, 0.0005, and 0.0001 au.

**Figure 4:**
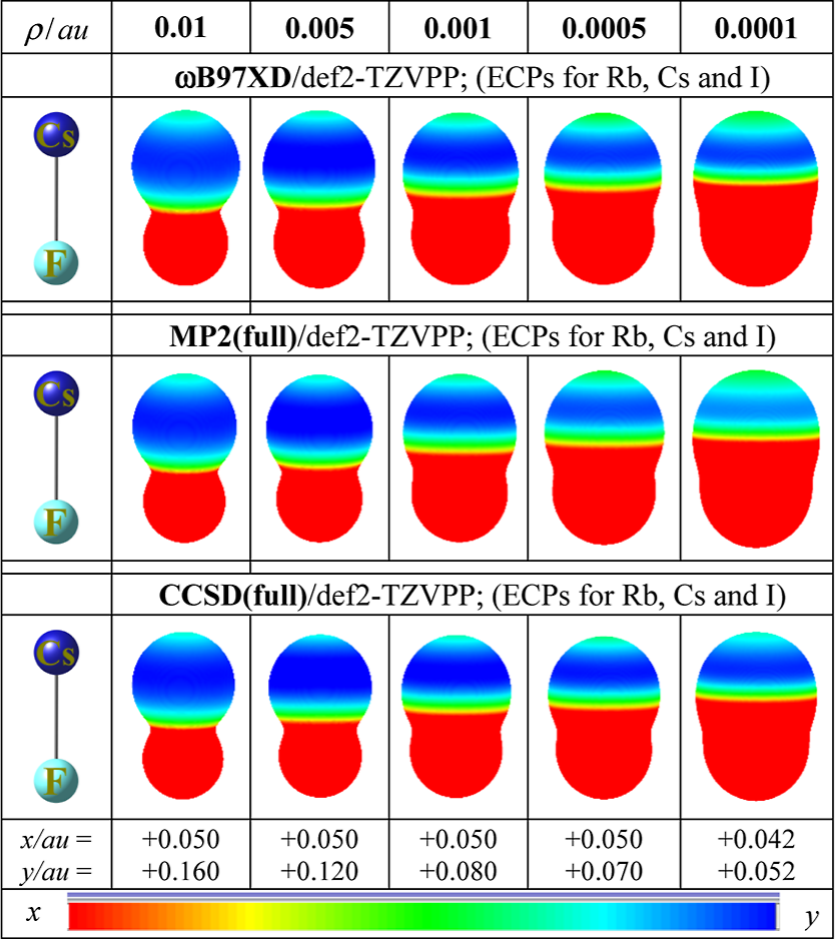
Computed CsF surface ESPs obtained at three different levels of theory and for five different isodensity surfaces (ρ = 0.01, 0.005, 0.001, 0.0005, and 0.0001 au), the same color range (*x* to *y*) is used for the iso-surfaces in each column. Going from left to right, *x* and *y* were selected for contrast to give a clear view of the positive band on the Cs surface.

That range of charge densities (a change of two orders magnitude (from 0.01 to 0.0001 au); Figure 4) spans the iso-surfaces that are typically selected and examined in the assessment of sigma holes and other characteristics of surface potentials associated with weak intermolecular interactions.^56,57^ The results in Figure 4 indicate that the ring phenomenon is continuous and stable indeed across a wide range of iso-surfaces and is by no mean restricted to the 0.001 au iso-surface, for instance, that is considered throughout this work.

In sum, the sigma ring phenomenon is independent of both basis set choice and the methods employed, and, as we will show shortly, is not without consequence for the structure and bonding of metal halide complexes.

### Consequences of Sigma Rings for Bonding

By classical expectations, an unconstrained noncovalent sigma hole type interaction between a base, Y, and a group 1 center bonded to an electron withdrawing fragment, R, is expected to produce a roughly linear R–M···Y complex. That is indeed what we observe in reported instances of so-called lithium^22–28^ and sodium^28,29^ bonded complexes, but how do the corresponding K, Rb, and Cs systems behave?

To probe that question, we considered heterodimers of the form F–M···Y, where Y = B–H, B–F, NH_3_, and NCl_3_. We considered the model borylene bases because we wanted a simple monovalent base with a small atomic substituent. That way, we hoped, secondary effects such as interactions between F in the F–M unit and Y, if Y is very bulky, would be avoided or minimized. NH_3_ is included as a common simple base, and the choice of NCl_3_ will be justified presently.

Figure 5 shows the fully optimized noncovalently bound species that were closest to the linear starting arrangement from which each was generated. The linear arrangement persisted for LiF–Y and NaF–Y pairs (Figure 5), but we failed to recover any such structure for the KF, RbF, and CsF complexes for which *V*_s_(pole) ≠ *V*_s,max_. For those systems, where *V*_s_(ring) = *V*_s,max_, the bases migrate in a way that allows the lone pair better access to the sigma ring.

**Figure 5:**
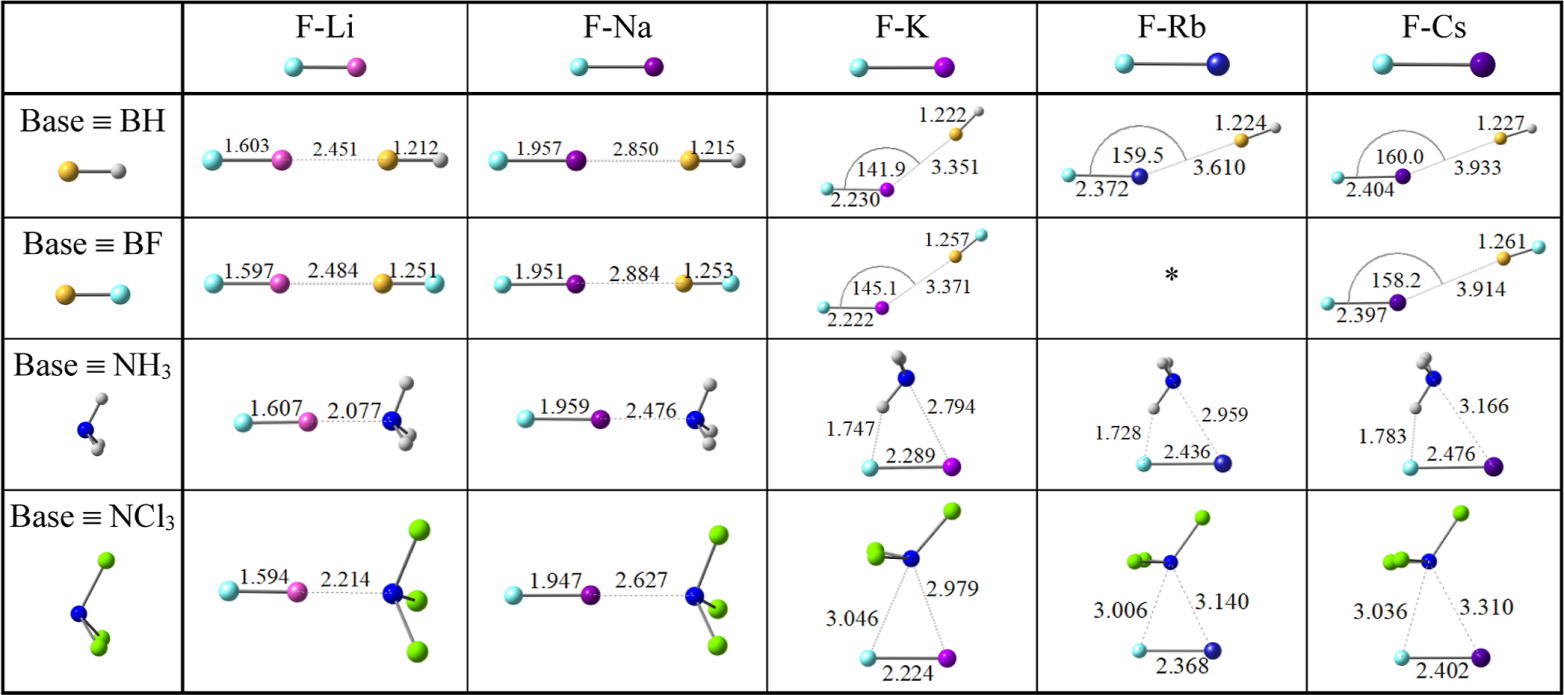
Weak sigma hole and other complexes between FM molecules and four simple bases. *Curiously, efforts to locate a simple noncovalent type complex for FRb···BF were repeatedly frustrated. Instead, a datively bonded species was consistently generated (see the Supporting Information).

For those K, Rb, and Cs systems, the F–M···Y angles are noticeably bent, and for better (for the stability of the structure) or worse (for any hope for simplicity), secondary interactions are introduced through F···H–N hydrogen bonding in F–M···NH_3_, for example. Prospects for hydrogen bonding are eliminated when Y = NCl_3_, but the preference for a bent geometry for M = K, Rb, and Cs is clear in that case as well. And, alas, secondary interactions always seem to find a way; replacing NH_3_ with NCl_3_ removes F···H–N hydrogen bonding but makes room for F···N–Cl pnictogen bonding (see the NH_3_ and NCl_3_ rows in Figure 5).^58–61^

We did not consider Y = NF_3_ since the polarizing power of the three F atoms makes NF_3_ a relatively poor base and an even better partner for pnictogen bonding. For the borylene (:BR) bases, two alternative outcomes, which readers might have anticipated since the B and M centers are so electron deficient, were observed: the oxidation of B­(I)­R to the trigonal planar B­(III)­RMF molecule or the formation of a covalent three membered F–M–B ring with the terminal R bonded to B (Figure S3)—a compromise on B­(III)­RMF that allows the F–M bond to maintain some fidelity. Interestingly, neither a linear nor a bent noncovalent complex was observed for FRb···BF. That system inexorably goes to the three-membered ring structure just mentioned (see Figure S3). It is unclear why RbF is exceptional in that way. BF is a weaker base than BH, however, so any noncovalent FM···BF complex is expected to produce, for any M, a shallower minimum on its potential energy surface compared to FM···BH, and to have easier access, thus, to alternative arrangements.

In sum, Figure 5 provides a great deal of conformational evidence that the presence of sigma rings (rather than sigma holes) on the M surfaces in the group 1 metal halides is quite consequential for structure and bonding.

### The Origin of Sigma Rings

How to account for the emergence of sigma rings on group one metal surfaces in these molecules? We have only discussed the fluorides so far, but we can report at this point that similar patterns are observed for all of the group 1 halides (see Table 3).

**Table 3:** Extrema in the Surface Electrostatic Potentials (in kcal·mol^–1^ units) at the Pole of M, *V*_s_(pole), and—Where It Exists—in a Latitudinal Belt on M, *V*_s_(ring), in MX Molecules, All Computed at the ωB97XD/def2-TZVPP and ωB97XD/dyall-acv3z Levels of Theory

		def2-TZVPP or ECP + valence basis set					all-electron dyall-acv3z				
		^a^Li	Na	K	Rb	Cs	Li	Na	K	Rb	Cs
F	*V*_s_(pole)	176.0	**131.1**	76.2	65.8	42.6	**182.3**	**130.8**	74.6	59.2	40.3
	*V*_s_(ring)	**176.8**	-	**77.7**	**67.9**	**50.1**	-	-	**76.2**	**62.5**	**46.7**
Cl	*V*_s_(pole)	**197.7**	**139.9**	89.0	78.6	58.5	**200.8**	**140.0**	88.7	73.8	56.9
	*V*_s_(ring)	-	-	**89.8**	**79.5**	**63.0**	-	-	**89.2**	**75.3**	**60.4**
Br	*V*_s_(pole)	**203.5**	**141.9**	92.3	81.5	62.2	**205.4**	**141.9**	91.7	76.8	60.6
	*V*_s_(ring)	-	-	**93.0**	**82.2**	**66.1**	-	-	**92.1**	**78.2**	**63.7**
I	*V*_s_(pole)	**209.4**	**143.5**	95.8	84.8	66.7	**210.8**	**143.9**	95.8	81.2	65.4
	*V*_s_(ring)	-	-	**96.4**	**85.3**	**70.0**	-	-	**96.1**	**82.4**	**68.0**

a For LiF, there is a very small ring around the pole of the atom, but the ring is so tight around the pole and the ESPs in the ring are so close to that at the pole as to be inconsequential. Such a ring arises on Li for LiF and LiCl with the all-electron x2c-TZVPP basis sets as well (see the Supporting Information), but not with the other basis sets considered.

Sigma rings arise on the K, Rb, and Cs centers for all of the halides considered. Whether we consider the *V*_s_(pole) or more limited *V*_s_(ring) values, however, the surface potentials on M decrease as we go from Li to Cs (across Table 3) and increase as we go from F to I (down Table 3). So CsF has the smallest *V*_s,max_ values (there *V*_s,max_ = *V*_s_(ring)) and LiI has the largest *V*_s,max_ values (there *V*_s,max_ = *V*_s_(pole)). Two of the all-electron basis sets that we considered (x2c-TZVPP and ANO-RCC-VTZP) in tandem with the ωB97XD method faithfully exhibit the qualitative trends just described except in one odd respect: *V*_s,max_ increase indeed going from MF to MBr, but the values suddenly decrease at the iodides (see the Supporting Information), a curious deviation that deserves exploration in another context.

Molecular surface potential maps generated using a common color scale (−0.10 au ≤ *V*_s_ ≤ +0.20 au) are presented in Figure 6 for the full slate of metal halides. Those ESP maps afford a fuller sense of the distribution of potentials across the whole molecular surface, reflect the variations in Table 3, and—since they are all on the same scale—can be compared with each other, and similar maps obtained using the MP2­(full) and CCSD­(full) methods are provided in Figures S4 and S5.

**Figure 6:**
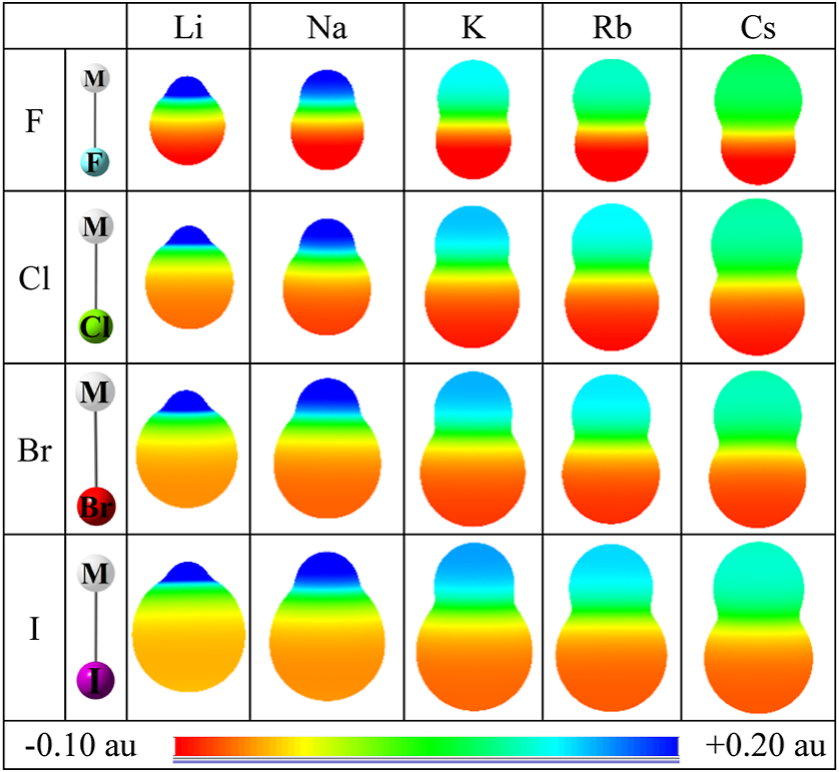
Computed surface potentials obtained at the ωB97XD/def2-TZVPP level for the group 1 metal halides.

Certain details, such as the sigma rings, are unfortunately not obvious at all with the color scale used in Figure 6. The benefit of using a common scale, however, is the chance to visualize the global trends established in Table 3, including certain remarkable patterns in the surface potentials that are independent of where *V*_s,max_ arises on the M surface.

We do not focus in this work on the negative surface potentials around the X centers, *V*_s_(X), in the MX molecules, but a perusal of the lower half of the ESP maps in Figure 6 attests to (i) a continuous decreases in |*V*_s_(X)| going from the fluoride to the iodide for each M (a fading of the red/orange surface going down Figure 6) and (ii) an increase in |*V*_s_(X)| going from Li to Cs for each X (an increasingly red surface going across Figure 6). So the F site in CsF will have the most negative, and I in LiI will have the least negative, *V*_s,min_ value among the group 1 metal halides.

Those outcomes (Figure 6) are well in line with conventional expectations; combining the very polarizing F and highly polarizable Cs atoms is expected to generate drastic Cs → F charge shifts and relatively large and negative *V*_s_(X) values, and the least polarizing of the halogens, I, is expected indeed to have its mildest effect on Li. So, the trends in *V*_s_(X) are rather unremarkable, even as the evolution of *V*_s_(M) (Table 3) defies simple explanations.

### Paradoxical Features and How to Explain Them

The observation that *V*_s,max_ on M in the M–X molecule increases as X gets less electronegative going from X = F to I (Table 3; notwithstanding the anomaly observed at I for certain basis sets, vide supra) is noteworthy. In Figure 6, that trend shows up as an increasingly blue M surface going down each column, which is difficult to discern for Li and Na since the surfaces are all intensely blue, but easier to see for K, Rb, and Cs.

One might expect *a priori* that *V*_s,max_ on M (Table 3) in any “MR” molecule and thus the potential across the whole M surface (Figure 6) would *decrease* (become less positive) as R becomes *less electronegative* (such as going from R = F to R = I, for instance). After all, that trend is observed repeatedly in the literature for sigma holes.^20^ For a halogen atom, X′, for instance, the sigma hole generated at X′ in the X′–F molecule fades if F is replaced successively by Cl, Br, and I, as the data we have assembled for X′–X molecules show in Table 4.

**Table 4:** Sigma Hole Maximum, *V*_s,max_, (in kcal·mol^–1^ units) Induced on X′ by a Halogen Atom, X, in an X′X Molecule for X′,X = F, Cl, Br, and I, All Computed at the ωB97XD/def2-TZVPP Level

	X′ = F	X′ = Cl	X′ = Br	X′ = I
F	13.8	41.5	50.9	59.4
Cl	–3.16	25.8	36.3	47.2
Br	–9.09	20.0	30.3	42.0
I	–16.8	11.4	21.4	33.6

Similarly, one expects that for such X′–X molecules, indeed for any X′–R system, the sigma hole on X′ will get larger and stronger (i.e., *V*_s,max_(X′) should *increase*) as X′ gets less electronegative, just as we find for X′–X going across each row in Table 4 from X′ = F to X′ = I.

But all of that is turned on its head when the halogen X′ atoms are replaced by the group 1 atoms! Compare the trends in Tables 3 and 4. As we noted above, *V*_s,max_(M) *increases* when F is replaced successively by Cl, Br, and I (down each column in Table 3), and it *decreases* as we go from M = Li to M = Cs (across each row in Table 3).

Importantly, those qualitative trends hold whether the surface maximum on M is at the pole or in a sigma ring. As is clear from Table 3, *V*_s_(M; pole) varies directly with *V*_s_(M; ring) in the K, Rb, and Cs halides, so the qualitative dependence of *V*_s_(M) on either X or M may be illustrated using *V*_s_(pole) only, which is available for Li through to Cs or in terms of *V*_s,max_ which would be *V*_s_(pole) in some cases and *V*_s_(ring) where the latter is present.

The contrast between the trends in the surface potentials on M in the group 1 M–X molecules (Table 3; Figure 6) and classical expectations (see data in Table 4 for X′–X) is brought into focus in Figure 7. The lines in (Figure 7a,b) show the trends in *V*_s_(M; pole) in the MX molecules (Figure 7a; cf. Table 3) and the sigma hole *V*_s,max_(X′) values for X′–X (Figure 7b; cf. Table 4). We chose *V*_s_(M; pole) for comparison with *V*_s,max_ for X′ because, *V*_s_(M; pole) exists for all of the metal halide molecules and, as we mentioned above (for cases where *V*_s_(M; ring) exists), *V*_s_(M; pole) varies directly with *V*_s_(M; ring). For easy comparison, the lines in Figure 7 are colored according to the row in the periodic table to which M and X′ belong, so the lines for M = Li (Figure 7a) and X′ = F (Figure 7b), for instance, have the same color, illustrating the failure of periodicity to unite these trends.

**Figure 7:**
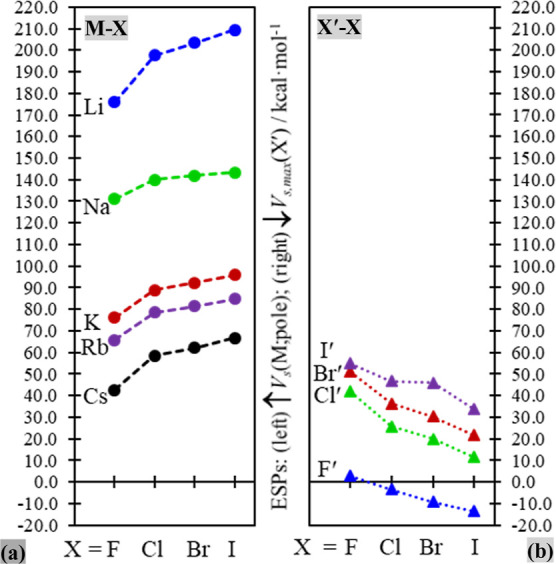
(a) *V*_s_(M; pole) values for M in M–X and (b) *V*_s,max_(X′) in the sigma hole for X′ in X′–X.

One aspect of our electrostatic potential data that is emphasized in Figure 7 is the differences in the magnitudes of *V*_s_(M) and *V*_s_(X′) induced by the halogen atoms to which they are bonded. For each X, the potentials are much more positive for the group 1 M atoms compared to the more electronegative X′ atoms, even as the trends in the MX and X′X data (see Figure 7) run in a way counter to each other.

### Dependence on M

To rationalize the contrarian patterns identified in *V*_s_(M) in the MX molecules, one might view the polarized M center, M^δ+^, as a way point on a continuum for which the two extrema at least are readily accessible computationally, namely, the neutral M atom and the monocation, M^+^. That perspective, we considered, would be particularly relevant in rationalizing key trends in *V*_s_(M) for the group 1 dihalides, since there is only one election (ns^1^) in the valence shells of group 1 atoms. We hypothesized that the surface potentials about the M center will vary directly with the extent to which that electron density from that ns^1^ orbital is stripped away from the pole over the surface of M toward the overlap region upon bonding to the X atom, especially since—unlike the halogens, and any other main group atom in fact—there is no other valence electron(s) to be redistributed across the atomic surface during the bonding process.

To probe that proposition, we computed and compared the surface potentials for the neutral ground state (doublet) group 1 atoms and their ground state singlet M^+^ cations and compared them with the *V*_s,max_ values for the M centers in the MF and MI molecules (see Table 5). The MF and MI species were selected since they span the largest and smallest *V*_s_(M) values (see plot on the left in Figure 7).

**Table 5:**
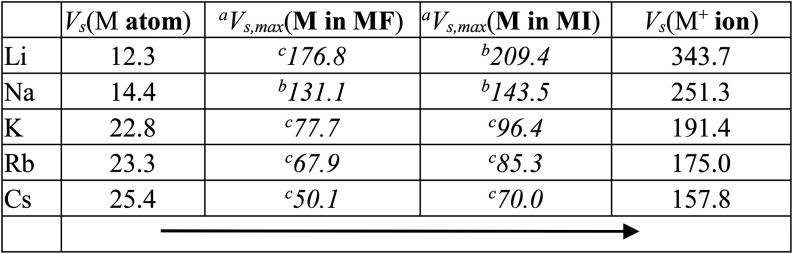
ωB97XD/def2-TZVPP Surface Potentials in kcal·mol^–1^ units for Group 1 Metal, M, (Doublet) Atoms, the Computed M Potentials in the MF and MI Compounds (Where *V*_s_(M) in the MX Molecules are Smallest and Largest, Respectively),^a^,^b^,^c^ and the Corresponding M^+^ (Singlet) Cation

a For each of the group 1 halides, the largest *V*_s_ values for each M among the MX halides is obtained typically when X = I and the smallest when X = F.

b For Li (except in LiF) and Na in their MX molecules, *V*_s,max_(M) = *V*_s_(M; pole).

c For Li in LiF and K, Rb, and Cs in their MX molecules, *V*_s,max_(M) = *V*_s_(M; ring).

The surface potentials for the free M atoms (see Table 5) are all relatively small, but they *increase* continuously going from M = Li to M = Cs such that *V*_s_(Cs^0^ atom) > 2 × *V*_s_(Li^0^ atom)! Once the single valence ns electron is removed, however, the situation is altered dramatically; *V*_s_ increases by 2 orders of magnitude, and disproportionately so, such that the trend is completely reversed relative to the M^0^ case: for the cations, *V*_s_(M^+^) *decreases* continuously (and quite rapidly at the first few steps) going from M = Li to M = Cs such that *V*_s_(Li^+^ ion) > 2 × *V*_s_(Cs^+^ ion)!

Based on the reasoning outlined above, the *V*_s_(M) values for the MX molecules are expected to fall between *V*_s_(M^0^) and *V*_s_(M^+^), and that is indeed the case (see Table 5). Moreover, the magnitudes of the *V*_s,max_(M) values for the MF and MI molecules exhibit the same trends as *V*_s_(M^+^) in Table 5: *V*_s,max_(Li^+^) > *V*_s,max_(Na^+^) > *V*_s,max_(K^+^) > *V*_s,max_(Rb^+^) > *V*_s,max_(Cs^+^).

Those observations are consistent with—though they do not authenticate—our framework for thinking about the trends in *V*_s,max_(M) in the MX molecules. They offer we think some insight into the possible origins of the anomalous trends in *V*_s_(M). That is, the magnitudes and trends in *V*_s_(M) , given the accumulation of electron density from M into the overlap region (hence M–X bond formation), seem to be dictated primarily by the partially (however moderate or radical) deshielding of the M^+^ cationic core.

That interpretation allows us to account for the observed decrease in *V*_s,max_(M) as M gets larger (going across Table 3 and down Table 5) based on the analogous trends in *V*_s_(M^+^). It is true; the M atoms get larger and more polarizable going down group 1 from Li to Cs, but an important consequence of the polarization of M by any given X atom is the substantial unveiling of the M^+^ core , which seems to strongly influence if not dictate the trend in the resulting *V*_s,max_(M) values.

### Dependence on X

The reason for the paradoxical strengthening of *V*_s,max_(M) as X gets larger and less electronegative (going down Table 3 and from MF to MI in Table 5) is unclear. But that trend is quite consequential for bonding. For two different NR_3_ bases, for example (see Table 6), the Li···N distance in the XLi···NR_3_ complexes *get shorter and stronger* even as X gets larger and less electronegative, consistent with the observed strengthening of *V*_s,max_(M) in that same direction.

**Table 6:** Li···N Distance (*r*), and BSSE Adjusted Binding Energy with Zero-point Energy Corrections, Δ*E*_bind_^ZPE,BSSE^ = *E* ^ZPE^(complex) – (*E* ^ZPE^(XLi) + *E* ^ZPE^ (NR_3_)) + *E* ^BSSE^, for Certain Linear X–Li···NR_3_ Complexes in Angstrom (Å), and kcal·mol^–1^ Units, Respectively

	X–Li···NH_3_		X–Li···NCl_3_	
	*r*(Li···N)	Δ*E*_bind_^ZPE,BSSE^	*r*(Li···N)	Δ*E*_bind_^ZPE,BSSE^
F	2.079	–17.7	2.214	–7.6
Cl	2.050	–20.0	2.177	–8.9
Br	2.043	–20.7	2.164	–9.4
I	2.034	–21.4	2.151	–9.9

We include in Table 6 the zero-point energy corrected binding energies which we obtained by subtracting the sum of the energies of the isolated minimum energy structures of the XLi “acid” and NR_3_ “base” units from the corresponding energy of the acid–base complex (see caption of Table 5). Those binding energies were further adjusted by including computed counterpoise corrections to account for basis set superposition errors (BSSEs).^50,51^ The associated enthalpy and free energy values are included in the Supporting Information (Table S8), and the general increase in the stability of the complex as X gets less electronegative is reflected in those values as well.

We considered Li in this context not primarily because it is small and easy to handle computationally but because the LiX molecules have *V*_s,max_ values that are at or very close to the pole of the metal and forms linear complexes, unlike K, Rb, and Cs where bending and secondary interactions (see Figure 5) limit our ability to discretely assess the impact of sigma hole (or ring) strengths on noncovalent M···N bonding.

We posit that the mechanism accounting for the phenomenon in question—the increase in *V*_s,max_ for any M in the MX molecules as X gets less electronegative—is related to the increasing size of the X atoms going down group 17 from F to I. Even though electronegativity decreases going from F to I, it appears that the single bond-pair electron density in the M–X bonding orbital becomes less effective in veiling the M surface opposite the M–X overlap region as X gets larger.

Such a trend is evidently less likely to arise, however, in cases where the M atom that is bonded to and polarized by X has more than one valence electron. Whether that additional electron density is due to an unpaired electron (·M–X) or lone pair (:M–X), or bonds to other substituents (R_*x*_M–X), the formation of any “M–X” bond inevitably drives a reorganization of electron density over the surface of the M center. In the case of the X′–X dihalides, for instance, the equatorial band of lone pairs on X′ responds to the formation of the X′–X bond, and the trends in *V*_s_(X′) (Table 4, and Figure 7b) are surely influenced by a self-consistent reorganization of the electron density on X′ during the X′–X bond formation. For *V*_s_(M) in the group 1 dihalides, however, (Table 3 and Figure 7a), no additional valence electron(s) is on M to offset the impact of, or respond in any way to, M–X bond formation.

This interpretation finds support if we permit a minor diversion here to consider the group 2 monohalides and their cations. Considering only the Be and Ba halides as representative cases, we find that (see Table S9) there is no sigma hole on Be in neutral BeX. Instead, there is, appropriately, an ESP minimum at the pole of Be opposite the Be–X bond (where the unpaired electron is localized), and that minimum becomes more negative going from X = F to X = I. But the sets of BeX^+^ and BaX^+^ cations follow the trends observed here for group 1 metal halides: The positive potentials on the M surfaces increase going from the fluoride to the bromides (with a decline at I—a tendency seen herein, vide supra, with certain basis sets for group 1 halides as well)! Moreover, while each BeX^+^ species has a sigma hole on Be, as is generally the case for the lighter group 1 metal halides, sigma rings are observed on Ba for all of its halides. Even more intriguingly, perhaps, sigma holes are observed on the neutral barium halides, and, despite the extra electron on those radical species, *V*_s,max_(Ba) for the neutral Ba halides (unlike the Be cases) show the same general X dependence as the group 1 system.

### Insight from Partial Charges

As is well-known, global point changes do not say much about the sign or presence of local sigma holes (or rings, etc.) at any point on a molecular surface. For the diatomic group 1 metal halides, however, they do provide a direct measure of the charge shift in the M–X bond away from M, which is always the far more electropositive partner, toward the X atom. From chemical intuition alone, the net change, *q*, on any M center in an MX bond is expected to decrease going from F to I down group 17, and natural population analysis of the charge distribution in those compounds conform to standard expectations for M = Li and Na (see Table 7), but not so much for the heavier elements; *q* is effectively identical for M = K, Rb, and Cs, regardless of the identity of X.

**Table 7:** Computed Natural Bond Orbital (Natural Population Analysis) Charges on M in MX, *q*/|e|

	Li	Na	K	Rb	Cs
F	0.96	0.96	0.95	0.96	0.94
Cl	0.93	0.94	0.95	0.95	0.94
Br	0.92	0.93	0.95	0.96	0.95
I	0.89	0.91	0.95	0.95	0.95

Indeed, changing X hardly alters the point charges for any set of group 1 metal halides. The charges are all quite high in every case (Table 7), and even for Li, which is most sensitive to X, Δ*q*/|e| going from LiF to LiI is only 0.07! All of the group 1 halides are extremely polar, virtually ionic, species in the gas phase, so changes in the net charges are unhelpful for rationalizing the variations observed in the surface potentials. If anything, the data in Table 7 alerts us to the fact that at the extremes—at the most polar end of the spectrum of chemical bonding, especially here where M in the MX molecule is monovalent and always very polarizable—jumps in the electronegativity of X do not necessarily mean jumps in charge transfer.

### Insight from the MH Systems

An argument from atomic radii for explaining the increase in *V*_s_(M) as X gets larger receives support from a comparison of the *V*_s_(M) values for the metal hydrides, MH, with those for MX in Table 3. The (Pauling) electronegativity of *H* (χ­(*H*) = 2.20) is well below that of *F* (χ­(*F*) = 3.98), but revised estimates remind us that the ionic radii of the fluoride (*r* = 1.14 Å) and hydride (*r* = 1.20 Å) ions are comparable.^62^ And, remarkably, the *V*_s_(M) values for the group 1 hydrides—(*V*_s_(pole);*V*_s_(ring) = Li (193.6), Na (118.5), K (74.5; 78.6), Rb (65.5; 68.8), and Cs (44.4; 56.6) all in kcal·mol^–1^ units)—are comparable to those of the group 1 fluorides (see Table 3), rather than the iodides, for instance, even though χ­(*I*) = 2.66 is much closer to χ­(*H*).

### More on the Ring Phenomenon

All of the levels of theory that we have considered predict the presence of sigma rings on the larger M centers in group 1 metal halides. They all agree as well that the difference between the ESP values at the pole compared to the ring, Δ*V*_s(r–p)_, *increases* going from M = K to M = Cs for each X, and *decrease* as X gets larger (going down Table 3) even if different levels of theory disagree on the direction of the shift at X = I. So, although *V*_s,max_(Cs) in CsF is the smallest *V*_s,max_(M) value for group 1 metal halides, Δ*V*_s(r–p)_(M) is largest for CsF.

The reason for the emergence of sigma rings on the surfaces of group 1 metals is unclear, but it is helpful to keep in mind that expectations of the sigma hole phenomenon emerged from analyses of p-block atoms, especially terminal halides. Those atomic centers have (ns–np hybridized, or np) orbitals involved in bond formation that are distinguished in shape and nodal structure from the ns orbitals of group 1 metals. Since the ns orbital has no angular node, is cylindrically symmetric about the M–X bond axis, is singly occupied, and is completely exposed (unlike the np^1^ halogen orbital in halogen bonding that is engulfed by lone pairs), there is no reason for the maximum in the surface potential to be at the pole of M rather than at a somewhat lower latitude on the outer “hemisphere” of a very polarized M atomic center in the molecule. Preparation for M–X bond formation involves a flow of the ns electron density across the M surface into the M–X overlap region with no lone pair(s) or vicinal bonds on M to respond to the impact of the M → X charge shift on *V*_s_(M).

The ring phenomenon appears to arise from a confluence of different features of the M atomic center. The fact that M has one valence electron in a cylindrically symmetric valence orbital in the molecule is not a sufficient, if necessary, condition. A very high atomic softness or polarizability of M relative to the polarizing power of X appears to favor ring formation as well. Indeed, the odd presence of a small ring on Li in LiF and the absence of any such development in heavier lithium halides or the NaX molecules actually affirms a role for polarizability in ring formation since—against the general trends in the periodic table—the atomic polarizabilities of Li (α_Li_ = 164(0) au) is at the upper end of the error bar for Na (α_Na_ = 163(1) au),^63^ even as α_M_ increases monotonously for the rest of group 1: α_K_ = 290(1) au, α_Rb_ = 320(1) au, and α_Cs_ = 401(1) au.

The results in Table 3 suggest that increasing the electronegativity of the substituent (X) to which M is bonded favors ring formation on M and enhances Δ*V*_s(r–p)_(M), but bond polarity we find is neither necessary nor sufficient for sigma hole formation. A check of the group 1 dimers—the M_2_ homonuclear diatomics—uncovers a sigma ring on the Cs centers in Cs_2_ (Figure 8), while the other M_2_ systems possess conventional sigma holes around the poles of the M atoms.

**Figure 8:**
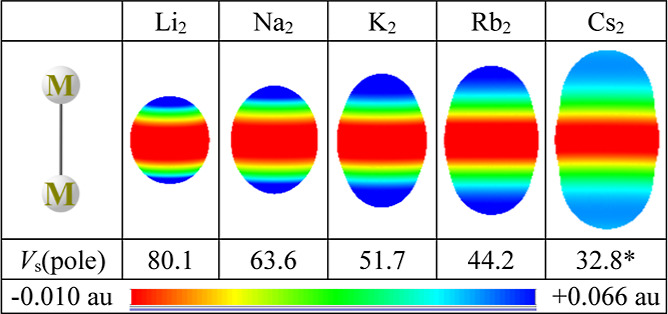
Computed surface potentials on the 0.001 au iso-surface obtained at the ωB97XD/def2-TZVPP level (all plotted on a common color scale of −0.010 to +0.066 au) for the M_2_ molecules. The associated *V*_s_(pole) values for the M sites in the molecules are included. *For M = Cs, V_s,max_ = *V*_s_(ring) = 34.8 kcal·mol^–1^. For all of the other cases, *V*_s,max_ = *V*_s_(pole). The global ESP minimum for the molecule (in the red region in the ESP maps) is a ring around the M–M bond.

The question of what is happening at the M centers at the level of individual valence and core atomic orbitals under the influence of polarization and M–X bond formation offers us more challenges for the future. The characterization of the sigma ring phenomenon and an understanding of certain conditions that foster its emergence have provided us with extraordinary insights into the compounds of those metals on the edge of the periodic table. There, several counterintuitive trends in the surface potentials arise that show a complex dependence on valence shell electron configuration, atomic size, and polarizability.

## Summary and Conclusions

Chemical intuition is not always reliable, even if it is often useful. That is especially true on the margins, at extremes. In a certain sense, the group 1 elements are at such a limit in the periodic table. After all, if iodine is electronegative enough to achieve a charge transfer of 0.9e or more in the halides of monovalent group 1 metals, there is no room left for fluorine with its much larger electronegativity to do much better. Studying the extremes is vital, however, since the unusual conditions that tend to obtain there tend to generate fascinating phenomena, a category to which the sigma rings that we describe and examine herein arguably belong. Those latitudinal zones or frusta are regions of high positive potential that arise in the surface electrostatic potentials around certain terminal group 1 metal atomic centers in their halide (MX), hydride (MH), and even homonuclear (M_2_) diatomic molecules. Like sigma holes that are known to arise in terminal halides, for instance, sigma rings have significant structural consequences for noncovalent complexes involving group 1 metal atoms.

Additionally, we find that variations in the surface potentials at M in the group 1 metal halides (MX) (whether the M site possesses a sigma hole or ring) run contrary to classical expectations, which have been shaped largely by observations on terminal group 17 atoms.

Conditions necessary for the emergence of sigma rings are discussed, affording us some bases for the prediction of or accounting for the lack of such features on the electrostatic potential surfaces of other terminal atomic centers. The sigma ring phenomenon is shown to be stable with respect to the choice of modern basis sets, and methods, and with respect to the choice of isodensity surface within a wide range of electron densities. This work opens up too the question of how to decisively and explicitly trace the orbital origins of the sigma ring phenomenon.

## Supplementary Material





## Notes

Additional computational data, including computed sigma hole and sigma ring extrema in the surface potentials obtained at several different levels of theory, graphical representations of relevant surface potentials, geometrical and thermodynamic quantities, details of basis sets used, and a set of “.xyz” files with viewable Cartesian coordinates (ZIP)
Figures and Tables (PDF)

## References

[1] Dumas J. M., Geron C., Peurichard H., Gomel M. (1976). MX_4_-Organic
Base Interactions (M = C, Si; X = Cl, Br). Study of the Influence
of the Central Element and the Halogen. Bull.
Soc. Chim. Fr..

[2] Dumas J. M., Kern M., Janier-Dubry J. L. (1976). Cryoscopic and Calorimetric Study
of MX_4_-Polar Organic Base Interactions (M = C, SI, X =
Cl, Br)-Influence of Element and of Halogen. Bull. Soc. Chim. Fr..

[3] Brinck T., Murray J. S., Politzer P. (1992). Surface Electrostatic Potentials
of Halogenated Methanes as Indicators of Directional Intermolecular
Interactions. Int. J. Quantum Chem..

[4] Dumas J. M., Peurichard H., Gomel M. (1978). CX_4_...Base Interactions
as Models of Weak Charge-Transfer Interactions: Comparison with Strong
Charge-Transfer and Hydrogen-Bond Interactions. J. Chem. Res..

[5] Clark T., Hennemann M., Murray J. S., Politzer P. (2007). Halogen Bonding: The
σ-Hole. J. of Mol. Model..

[6] Clark T. (2013). σ-Holes. WIREs Comput. Mol. Sci..

[7] Wittmaack B. K., Crigger C., Guarino M., Donald K. J. (2011). Charge Saturation
and Neutral Substitutions in Halomethanes and Their Group 14 Analogues. J. Phys. Chem. A.

[8] Martin D. R. (1948). Coordination
Compounds of Boron Bromide and Boron Iodide. Chem. Rev..

[9] Brammer L., Bruton E. A., Sherwood P. (2001). Understanding
the Behavior of Halogens
as Hydrogen Bond Acceptors. Cryst. Growth Des..

[10] Donald K. J., Wittmaack B. K., Crigger C. (2010). Tuning σ-Holes: Charge Redistribution
in the Heavy (Group 14) Analogues of Simple and Mixed Halomethanes
Can Impose Strong Propensities for Halogen Bonding. J. Phys. Chem. A.

[11] Politzer P., Murray J. S., Lane P., Concha M. C. (2009). Electrostatically
Driven Complexes of SiF_4_ with Amines. Int. J. Quantum Chem..

[12] Murray J.
S., Lane P., Politzer P. (2009). Expansion of the Sigma-Hole Concept. J. Mol. Model..

[13] Zahn S., Frank R., Hey-Hawkins E., Kirchner B. (2011). Pnicogen Bonds: A New
Molecular Linker?. Chem.—Eur. J..

[14] Donald K. J., Tawfik M. (2013). The Weak Helps the Strong: Sigma-Holes
and the Stability
of MF_4_·Base Complexes. J. Phys.
Chem. A.

[15] Grabowski S. J. (2014). Tetrel
Bond-σ-Hole Bond as a Preliminary Stage of the S_N_2 Reaction. Phys. Chem. Chem. Phys..

[16] Scilabra P., Terraneo G., Resnati G. (2019). The Chalcogen Bond in Crystalline
Solids: A World Parallel to Halogen Bond. Acc.
Chem. Res..

[17] Brock D. S., Bilir V., Mercier H. P. A., Schrobilgen G. J. (2007). XeOF_2_, F_2_OXeN≡CCH_3_, and XeOF_2_·nHF: Rare Examples of Xe­(IV) Oxide
Fluorides. J. Am. Chem. Soc..

[18] Bauzá A., Frontera A. (2015). Aerogen Bonding Interaction: A New Supramolecular Force?. Angew. Chem., Int. Ed..

[19] Alkorta I., Elguero J., Frontera A. (2020). Not Only Hydrogen Bonds: Other Noncovalent
Interactions. Crystals.

[20] Donald, K. J. Sigma Hole Supported Interactions: Qualitative Features, Various Incarnations, and Disputations (Chapter 11). In Exploring Chemical Concepts Through Theory and Computation; Liu, S. , Ed.; John Wiley & Sons, 2024; pp 285–316.

[21] Scerri E. (2008). The Role of
Triads in the Evolution of the Periodic Table: Past and Present. J. Chem. Educ..

[22] Shigorin D. N. (1959). Infra-Red
Absorption Spectra Study of H-Bonding and of Metal-Element Bonding. Spectrochim. Acta.

[23] Kollman P. A., Liebman J. F., Allen L. C. (1970). Lithium
Bond. J. Am. Chem. Soc..

[24] Latajka Z., Scheiner S. (1984). Ab Initio Comparison of H Bonds and Li Bonds. Complexes
of LiF; LiCl, HF, and HCl with NH_3_. J. Chem. Phys..

[25] Szczȩśniak M. M., Latajka Z., Piecuch P., Ratajczak H., Orville-Thomas W. J., Rao C. N. R. (1985). Theoretical Studies of Lithium Bonding
in Lithium Chloride/Aliphatic Amine Complexes. Chem. Phys..

[26] Sannigrahi A. B. (1986). The Lithium
Bond. J. Chem. Educ..

[27] Sannigrahi A., Kar T., Niyogi B. G., Hobza P., Schleyer P. v R. (1990). The Lithium Bond
Reexamined. Chem. Rev..

[28] Das A., Arunan E. (2022). Non-Covalent Bonds in Group 1 and Group 2 Elements:
The Alkalene Bond. Phys. Chem. Chem. Phys..

[29] Esrafili M. D., Mohammadirad N. (2014). Halogen Bond Interactions Enhanced by Sodium Bonds
- Theoretical Evidence for Cooperative and Substitution Effects in
NCX···NCNa···NCY Complexes (X = F, Cl,
Br, I; Y = H, F, OH). Can. J. Chem..

[30] Frisch, M. J. ; Trucks, G. W. ; Schlegel, H. B. ; Scuseria, G. E. ; Robb, M. A. ; Cheeseman, J. R. ; Scalmani, G. ; Barone, V. ; Petersson, G. A. ; Nakatsuji, H. ; Li, X. ; Caricato, M. ; Marenich, A. V. ; Bloino, J. ; Janesko, B. G. ; Gomperts, R. ; Mennucci, B. ; Hratchian, H. P. ; Ortiz, J. V. ; Izmaylov, A. F. ; Sonnenberg, J. L. ; Williams-Young, D. ; Ding, F. ; Lipparini, F. ; Egidi, F. ; Goings, J. ; Peng, B. ; Petrone, A. ; Henderson, T. ; Ranasinghe, D. ; Zakrzewski, V. G. ; Gao, J. ; Rega, N. ; Zheng, G. ; Liang, W. ; Hada, M. ; Ehara, M. ; Toyota, K. ; Fukuda, R. ; Hasegawa, J. ; Ishida, M. ; Nakajima, T. ; Honda, Y. ; Kitao, O. ; Nakai, H. ; Vreven, T. ; Throssell, K. ; Montgomery, J. A. ; Peralta, J. E. ; Ogliaro, F. ; Bearpark, M. J. ; Heyd, J. J. ; Brothers, E. N. ; Kudin, K. N. ; Staroverov, V. N. ; Keith, T. A. ; Kobayashi, R. ; Normand, J. ; Raghavachari, K. ; Rendell, A. P. ; Burant, J. C. ; Iyengar, S. S. ; Tomasi, J. ; Cossi, M. ; Millam, J. M. ; Klene, M. ; Adamo, C. ; Cammi, R. ; Ochterski, J. W. ; Martin, R. L. ; Morokuma, K. ; Farkas, O. ; Foresman, J. B. ; Fox, D. J. Gaussian 16, Revision C.02; Gaussian, Inc.: Wallingford, CT, 2019.

[31] Weigend F., Ahlrichs R. (2005). Balanced Basis Sets
of Split Valence, Triple Zeta Valence
and Quadruple Zeta Valence Quality for H to Rn: Design and Assessment
of Accuracy. Phys. Chem. Chem. Phys..

[32] Weigend F. (2006). Accurate Coulomb-Fitting
Basis Sets for H to Rn. Phys. Chem. Chem. Phys..

[33] Chai J.-D., Head-Gordon M. (2008). Long-Range
Corrected Hybrid Density Functionals with
Damped Atom-Atom Dispersion Corrections. Phys.
Chem. Chem. Phys..

[34] Frisch M. J., Head-Gordon M., Pople J. A. (1990). A Direct MP2 Gradient Method. Chem. Phys. Lett..

[35] Head-Gordon M., Head-Gordon T. (1994). Analytic MP2 Frequencies without
Fifth-Order Storage.
Theory and Application to Bifurcated Hydrogen Bonds in the Water Hexamer. Chem. Phys. Lett..

[36] The “full” condition mandates that all (core and valence) electrons be included in correlation calculations.

[37] Čížek, J. On the Use of the Cluster Expansion and the Technique of Diagrams in Calculations of Correlation Effects in Atoms and Molecules. In Advances in Chemical Physics; Lefebvre, R. , Moser, C. , Eds.; John Wiley & Sons, Ltd, 1969; Vol. XIV, pp 35–89.

[38] Scuseria G. E., Schaefer H. F. (1989). Is Coupled Cluster Singles and Doubles
(CCSD) More Computationally Intensive than Quadratic Configuration
Interaction (QCISD)?. J. Chem. Phys..

[39] Kendall R. A., Dunning T. H., Harrison R. J. (1992). Electron Affinities of the First-row
Atoms Revisited. Systematic Basis Sets and Wave Functions. J. Chem. Phys..

[40] Woon D. E., Dunning T. H. (1993). Gaussian Basis Sets for Use in Correlated Molecular
Calculations. III. The Atoms Aluminum through Argon. J. Chem. Phys..

[41] These effective core pseudopotentials and valence basis sets were retrieved from this. https://www.tc.uni-koeln.de/PP/clickpse.en.html (accessed June 12:2026).

[42] Lim I. S., Schwerdtfeger P., Metz B., Stoll H. (2005). All-Electron and Relativistic
Pseudopotential Studies for the Group 1 Element Polarizabilities from
K to Element 119. J. Chem. Phys..

[43] Peterson K. A., Shepler B. C., Figgen D., Stoll H. (2006). On the Spectroscopic
and Thermochemical Properties of ClO, BrO, IO, and Their Anions. J. Phys. Chem. A.

[44] Dyall K. G. (2012). Core Correlating
Basis Functions for Elements 31–118. Theor. Chem. Acc..

[45] Dyall K. G., Tecmer P., Sunaga A. (2023). Diffuse Basis Functions for Relativistic
s and d Block Gaussian Basis Sets. J. Chem.
Theory Comput..

[46] Pollak P., Weigend F. (2017). Florian Segmented Contracted
Error-Consistent Basis
Sets of Double- and Triple-ζ Valence Quality for One- and Two-Component
Relativistic All-Electron Calculations. J. Chem.
Theory Comput..

[47] Roos B. O., Veryazov V., Widmark P.-O. (2004). Relativistic
Atomic Natural Orbital
Type Basis Sets for the Alkaline and Alkaline-Earth Atoms Applied
to the Ground-State Potentials for the Corresponding Dimers. Theor. Chem. Acc..

[48] Roos B. O., Lindh R., Malmqvist P.-Å., Veryazov V., Widmark P.-O. (2004). Main Group
Atoms and Dimers Studied with a New Relativistic ANO Basis Set. J. Phys. Chem. A.

[49] Pritchard B. P., Altarawy D. B., Didier, Gibson T. D., Windus T. L. (2019). A New Basis
Set Exchange: An Open, Up-to-date Resource for the Molecular Sciences
Community. J. Chem. Inf. Model..

[50] Boys S. F., Bernardi F. (1970). Calculation of Small Molecular Interactions
by Differences
of Separate Total Energies - Some Procedures with Reduced Errors. Mol. Phys..

[51] Jensen, F. Introduction to Computational Chemistry, For a description of this procedure; Wiley: New York, 1999; pp 172–173.

[52] Dennington, R. ; Keith, T. A. ; Millam, J. M. , GaussView, 6.0; Semichem Inc., Shawnee Mission, KS, 2016.

[53] Chemcraft 1.8-Graphical Software for Visualization of Quantum Chemistry Computations. https://www.chemcraftprog.com (accessed June 12, 2026).

[54] Lu T., Chen F. (2012). Multiwfn: a multifunctional wavefunction analyzer. J. Comput. Chem..

[55] Lu T., Chen F. (2012). Quantitative analysis
of molecular surface based on improved Marching
Tetrahedra algorithm. J. Mol. Graphics Modell..

[56] Bader R. F. W., Carroll M. T., Cheeseman J. R., Chang C. (1987). Properties of Atoms
in Molecules: Atomic Volumes. J. Am. Chem. Soc..

[57] Murray J. S., Politzer P. (2011). The Electrostatic Potential: An Overview. WIREs Comput. Mol. Sci..

[58] Murray J. S., Lane P., Politzer P. (2007). A Predicted
New Type of Directional
Noncovalent Interaction. Int. J. Quantum Chem..

[59] Scheiner S. (2011). Effects of
Substituents upon the P···N Noncovalent Interaction:
The Limits of Its Strength. J. Phys. Chem. A.

[60] Brzeski J. (2022). On the Influence
of Pnictogen Bonding on Acidity. Polyhedron.

[61] Ouyang L., Luo R., Lu Y., Xu Z., Zhu W. (2025). Pnictogen Bonds between
Electrophilic Pnictogens and Nucleophilic Metal Centers: A Combined
Database Survey and Theoretical Investigation. J. Phys. Chem. A.

[62] Pyykkö P., Atsumi M. (2009). Molecular Single-Bond
Covalent Radii for Elements 1–118. Chem.—Eur.
J..

[63] Schwerdtfeger P., Nagle J. K. (2019). 2018 Table of Static
Dipole Polarizabilities of the
Neutral Elements in the Periodic Table. Mol.
Phys..

