# Supplementary material for: The Sigma Ring and Other Distinctive Features of Surface Potentials of Group 1 Systems
Source: J Phys Chem A. 2026 Jul 8;130(28):5445–55. doi: 10.1021/acs.jpca.6c02529 (PMC13383737; doi:10.1021/acs.jpca.6c02529)
Supplement: Supplementary file 2 [file jp6c02529_si_002.pdf]

The Sigma Ring and Other Distinctive Features of Surface Potentials of Group 1 Systems

Kelling J. Donald* Victoria Rankin, Brice Di Carlo, Jonathan E. Findley, and Ethan B. Leonard

Department of Chemistry, Gottwald Center for the Sciences, University of Richmond, Richmond, Virginia 23173, United States

Supporting Information

* Corresponding author. K. J. Donald, Tel.: 1-804-484-1628. E-mail: kdonald@richmond.edu ORCID: 0000-0001-9032-4225

Table of Contents

Abbreviated Captions*	Page
Guide to *.xyz* Files	S3
Note on Basis Set Information	S3
**Table S1**: Ring and pole extrema on M from computed group 1 metal halide ESP data using the ωB97XD method and the aug-cc-pVTZ basis sets or Stuttgart-Cologne ECPs and valence basis sets (for K, Rb, Cs, and I).	S3
**Table S2**: Ring and pole extrema on M from computed group 1 metal halide ESP data using the ωB97XD method and the def2-TZVPP basis sets as defined in Gaussian 16, which employs basis sets for Rb, Cs, and I.	S3
**Table S3**: Ring and pole extrema on M from computed group 1 metal halide ESP data using the ωB97XD method and the dyall-acv3z basis sets	S4
**Table S4**: Ring and pole extrema on M from computed group 1 metal halide ESP data using the ωB97XD method and the x2c-TZVPPall basis sets	S4
**Table S5**: Ring and pole extrema on M from computed group 1 metal halide ESP data using the ωB97XD method and the ANO-RCC-VTZP basis sets	S4
**Table S6**: Ring and pole extrema on M from computed group 1 metal halide ESP data using the MP2(full) method and the def2-TZVPP basis sets.	S5
**Table S7**: Ring and pole extrema on M from computed group 1 metal halide ESP data using the CCSD(full) method and the def2-TZVPP basis sets.	S5
**Table S8:** Computed Li···N distances and basis set superposition error corrected binding energies (with zero-point energies), enthalpies, and free energies for certain linear XLi···NR_3 complexes.	S6
**Table S9:** Ring and pole extrema on M from computed surface ESP data for Be and Ba metal halide molecules and cations using the ωB97XD method and the def2-TZVPP basis sets.	S6
**Figure S1**: Computed ESPs (on the 0.001 au iso-surface) for group 1 fluorides, all on the same color scale.	S7
**Figure S2**: Computed ESPs (on the 0.001 au iso-surface) for group 1 fluorides, each on a molecule-specific color scale emphasizing sigma rings.	S8
**Figure S3**: Alternative, more covalent, structures obtained for the group 1 fluorides and BR bases (R = H, F) starting from a triangular starting structure that was found to be a minimum for CsF.	S9
**Figure S4**: Computed surface potentials (on the 0.001 au iso-surface) obtained at the MP2(full)/def2-TZVPP level for the group 1 metal halides.	S9
**Figure S5**: Computed surface potentials (on the 0.001 au iso-surface) obtained at the CCSD(full)/def2-TZVPP level for the group 1 metal halides.	S10

*More detailed captions are provided below for the individual figures and tables.

Guide to .xyz files

A set of *.xyz* files containing the optimized coordinates for the isolated MX monomers and various complexes is included as part of the supporting information.

Note on basis set information

The basis sets used in this work are included in a separate file as part of the supporting information.

TABLES

Table S1: Ring and pole extrema on M in the computed group 1 metal halide (MX) electrostatic potentials (in kcal·mol^{-1} units,* on the 0.001 au iso-surface) obtained using the ωB97XD method and the aug-cc-pVTZ basis sets or Stuttgart-Cologne ECPs and valence basis sets (for K, Rb, Cs, and I).

		aug-cc-pVTZ or ECP + valence basis set				
		Li-X	Na-X	K-X	Rb-X	Cs-X
F	V_s(pole)	**180.2**	**136.8**	*75.3*	*58.1*	*42.4*
	V_s(ring)	-	-	**76.5**	**61.6**	**49.1**
Cl	V_s(pole)	**200.8**	**144.9**	*88.8*	*72.4*	*58.2*
	V_s(ring)	-	-	**89.0**	**73.9**	**62.0**
Br	V_s(pole)	**205.2**	**146.2**	*91.7*	*75.3*	*61.6*
	V_s(ring)	-	-	**91.8**	**76.6**	**64.9**
I	V_s(pole)	**210.3**	**147.2**	**90.0**	*78.4*	65.2
	V_s(ring)	-	-	[a] **-**	**79.6**	**68.1**

* Conversion factors: 1 kcal·mol^{-1} = 4.336411 × 10^{-2} eV = 1.593601 × 10^{-3} au.

[a] For K-I, there is a very small ring around the pole of the atom but the ring is so tight around the pole, and the ESPs in the ring agree so closely (to three significant figures) to that at the pole as to be inconsequential.

Table S2: Ring and pole extrema on M in the computed group 1 metal halide (MX) electrostatic potentials (in kcal·mol^{-1} units, on the 0.001 au iso-surface) obtained using the ωB97XD method and the def2-TZVPP basis sets as defined in Gaussian 16, which employs basis sets for Rb, Cs, and I.

		def2-TZVPP or ECP + valence basis set				
		Li-F	Na-F	K-F	Rb-F	Cs-F
F	V_s(pole)	*176.0*	**131.1**	*76.2*	*65.8*	*42.6*
	V_s(ring)	**176.8**	-	**77.7**	**67.9**	**50.1**
Cl	V_s(pole)	**197.7**	**139.9**	89.0	*78.6*	*58.5*
	V_s(ring)	-	-	**89.8**	**79.5**	**63.0**
Br	V_s(pole)	**203.5**	**141.9**	*92.3*	*81.5*	*62.2*
	V_s(ring)	-	-	**93.0**	**82.2**	**66.1**
I	V_s(pole)	**209.4**	**143.5**	*95.8*	*84.8*	*66.7*
	V_s(ring)	-	-	**96.4**	**85.3**	**70.0**

Table S3: Ring and pole extrema on M in the computed group 1 metal halide (MX) electrostatic potentials (in kcal·mol^{-1} units, on the 0.001 au iso-surface) obtained using the ωB97XD method and the dyall-acv3z basis sets.

		All Electron dyall-acv3z				
		Li	Na	K	Rb	Cs
F	V_s(pole)	**182.3**	**130.8**	*74.6*	*59.2*	*40.3*
	V_s(ring)			**76.2**	**62.5**	**46.7**
Cl	V_s(pole)	**200.8**	**140.0**	88.7	*73.8*	*56.9*
	V_s(ring)			**89.2**	**75.3**	**60.4**
Br	V_s(pole)	**205.4**	**141.9**	*91.7*	*76.8*	*60.6*
	V_s(ring)			**92.1**	**78.2**	**63.7**
I	V_s(pole)	**210.8**	**143.9**	*95.8*	*81.2*	*65.4*
	V_s(ring)			**96.1**	**82.4**	**68.0**

Table S4: Ring and pole extrema on M in the computed group 1 metal halide (MX) electrostatic potentials (in kcal·mol^{-1} units, on the 0.001 au iso-surface) obtained using the ωB97XD method and the x2c-TZVPPall basis sets.

		All Electron x2c-TZVPPall				
		Li	Na	K	Rb	Cs
F	V_s(pole)	*175.8*	**131.0**	*75.9*	*57.0*	*37.3*
	V_s(ring)	**176.7**	-	**77.4**	**60.8**	**44.4**
Cl	V_s(pole)	*196.6*	**139.3**	*88.4*	*73.0*	*53.8*
	V_s(ring)	**196.9**	-	**89.1**	**74.3**	**57.9**
Br	V_s(pole)	**202.5**	**140.6**	*91.0*	*76.4*	*57.2*
	V_s(ring)	**-**	-	**91.6**	**77.2**	**60.9**
I	V_s(pole)	**203.7**	**135.1**	*83.4*	*71.3*	*43.2*
	V_s(ring)	**-**	**-**	**85.2**	**73.5**	**52.5**

Table S5: Ring and pole extrema on M in the computed group 1 metal halide (MX) electrostatic potentials (in kcal·mol^{-1} units, on the 0.001 au iso-surface) obtained using the ωB97XD method and the ANO-RCC-VTZP basis sets.

		All Electron ANO-RCC-VTZP				
		Li	Na	K	Rb	Cs
F	V_s(pole)	**191.9**	**136.0**	*80.1*	*61.5*	*42.6*
	V_s(ring)			**80.5**	**64.4**	**47.3**
Cl	V_s(pole)	**205.5**	**143.3**	*92.9*	*76.4*	*59.1*
	V_s(ring)			**93.0**	**77.6**	**62.1**
Br	V_s(pole)	**207.6**	**143.6**	*94.5*	*78.5*	*62.6*
	V_s(ring)			**94.6**	**79.5**	**65.3**
I	V_s(pole)	**173.7**	**126.1**	*72.9*	*55.5*	*43.7*
	V_s(ring)			**76.4**	**63.9**	**52.9**

Table S6: Ring and pole extrema on M in the computed group 1 metal halide (MX) electrostatic potentials (in kcal·mol^{-1} units, on the 0.001 au iso-surface) obtained at the **MP2(full)**/def2-TZVPP levels of theory.

		**MP2(full)**/def2-TZVPP or ECP + valence basis set				
		Li	Na	K	Rb	Cs
F	V_s(pole)	*175.0*	**129.0**	*76.9*	*65.1*	*43.0*
	V_s(ring)	**176.0**	-	**77.6**	**66.8**	**49.9**
Cl	V_s(pole)	**196.4**	**136.6**	*88.0*	*76.8*	*57.9*
	V_s(ring)	-	-	**88.5**	**77.7**	**62.2**
Br	V_s(pole)	**202.5**	**138.3**	*91.0*	*79.6*	*61.4*
	V_s(ring)	-	-	**91.4**	**80.3**	**65.2**
I	V_s(pole)	***208.3**	**139.5**	*94.0*	*82.7*	*65.5*
	V_s(ring)	-	-	**94.4**	**83.3**	**68.6**

*Two numerically close potentials were identified as maxima very close to the pole ((208.28 + 208.24)/2 = 208.3) / kcal·mol^{-1}. Such situations tend to arise in the (Multiwfn) ESP analysis if the ESP changes very gradually about the pole, or if the ring and V_s(ring) are close physically and numerically, respectively, to the pole and V_s(pole).

Table S7: Ring and pole extrema on M in the computed group 1 metal halide (MX) electrostatic potentials (in kcal·mol^{-1} units, on the 0.001 au iso-surface) obtained at the **CCSD(full)**/def2-TZVPP levels of theory.

		**CCSD(full)**/def2-TZVPP or ECP + valence basis set				
		Li	Na	K	Rb	Cs
F	V_s(pole)	*175.0*	**129.1**	*77.5*	*65.2*	*44.1*
	V_s(ring)	**175.8**	-	**78.1**	**66.8**	**50.6**
Cl	V_s(pole)	***196.2**	**137.0**	*88.7*	*77.1*	*59.1*
	V_s(ring)	-	-	**89.1**	**77.9**	**63.0**
Br	V_s(pole)	**202.2**	**138.7**	*91.6*	*79.8*	*62.5*
	V_s(ring)	-	-	**91.9**	**80.5**	**66.0**
I	V_s(pole)	**208.0**	**140.0**	*94.7*	*83.0*	*66.8*
	V_s(ring)	-	-	**95.0**	**83.5**	**69.6**

*Two numerically very close potentials were identified as maxima close to the pole ((196.22+ 196.20)/2 = 196.2) / kcal·mol^{-1}. Such situations tend to arise in the (Multiwfn) ESP analysis if the ESP changes very gradually about the pole, or if the ring and V_s(ring) are close physically and numerically, respectively, to the pole and V_s(pole).

Table S8: Li···N distances and BSSE adjusted binding energies with zero-point energy corrections, $\Delta E_{bind}^{ZPE,BSSE}$, and the corresponding enthalpy and free energy changes, $\Delta H_{bind}^{ZPE,BSSE}$ and $\Delta G_{bind}^{ZPE,BSSE}$ (for temperature, T, = 298.15 K), for linear X-Li···NR_3 complexes in angstrom (Å), and kcal·mol^{-1} units, respectively. The results were obtained using the ωB97XD method and the def2-TZVPP basis sets (employing ECPs and valence basis sets for Rb, Cs, and I).

	**R(Li···N)**	$\Delta E_{bind}^{ZPE,BSSE}$	$\Delta H_{bind}^{ZPE,BSSE}$	$\Delta G_{bind}^{ZPE,BSSE}$
	**X-Li···NH_3**			
**F**	2.079	-17.7	-18.0	-11.6
**Cl**	2.050	-20.0	-20.3	-13.8
**Br**	2.043	-20.7	-21.0	-14.4
**I**	2.034	-21.4	-21.7	-15.2
	**X-Li···NCl_3**			
**F**	2.214	-7.6	-7.3	-0.8
**Cl**	2.177	-8.9	-8.5	-2.0
**Br**	2.164	-9.4	-9.0	-2.7
**I**	2.151	-9.9	-9.5	-3.3

Table S9: Extrema in the surface electrostatic potentials (in kcal·mol^{-1} units) at the Be and Ba sites of the group 2 monohalides (·MX) and their (MX^+) cations on the 0.001 au iso-surface.* For neutral Be monohalides, those extrema are minima along the extension of the M-X bond axis. For the other cases, the extrema are sigma hole or sigma ring maxima on M. The results were obtained using the ωB97XD method and the def2-TZVPP basis sets (employing ECPs and valence basis sets for Rb, Cs, and I).

	**Neutral (doublet) MX Molecule**							**MX^+ (singlet) Cation**		
	**Be**			**Ba**					**Be**	**Ba**
**F**	$V_{s,\min}$	-0.32	**F**	V_s(pole)	**27.0**		**F**	V_s(pole)	**596.7**	*185.8*
				V_s(ring)	-			V_s(ring)	-	**206.6**
**Cl**	$V_{s,\min}$	-1.71	**Cl**	V_s(pole)	**39.0**		**Cl**	V_s(pole)	**621.2**	*188.8*
				V_s(ring)	-			V_s(ring)	-	**212.1**
**Br**	$V_{s,\min}$	-1.40	**Br**	V_s(pole)	**41.0**		**Br**	V_s(pole)	**616.3**	*188.5*
				V_s(ring)	-			V_s(ring)	-	**212.5**
**I**	$V_{s,\min}$	-1.79	**I**	V_s(pole)	**44.9**		**I**	V_s(pole)	**583.9**	*186.4*
				V_s(ring)	-			V_s(ring)	-	**212.2**

* Here we are considering extrema in the surface electrostatic potentials at the M sites only, whether they are minima or maxima, positive or negative. Extrema on the X sites are not considered here.

FIGURES

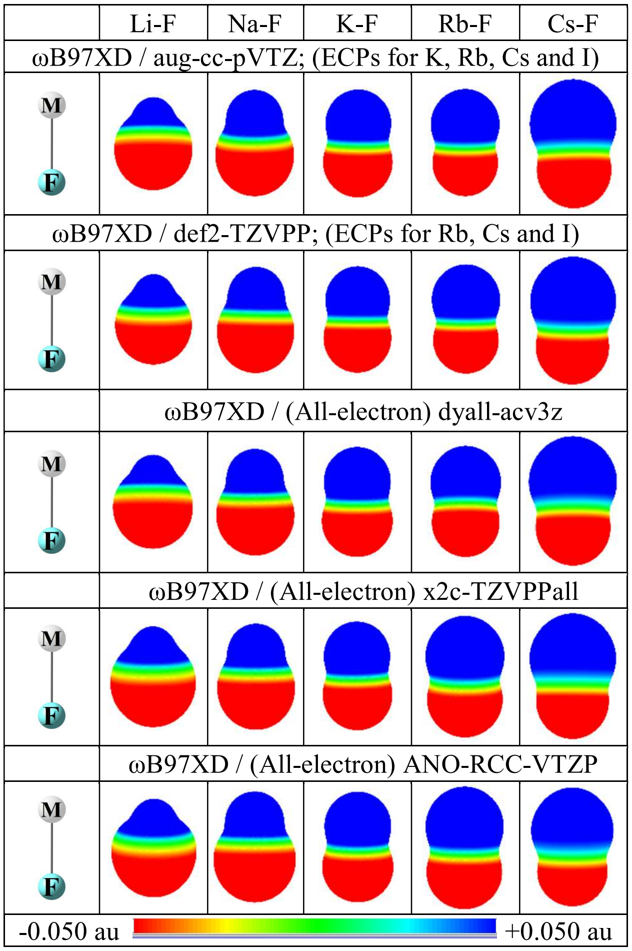

Figure S1: *Computed ESPs (on the 0.001 au iso-surface) for group 1 fluorides, all on the ESP color scale indicated. For each map, the MX molecule is oriented with M at the top as shown in the left column. Note: for electric potential 1au =* $E_h \cdot e^{-1}$*; for charge density 1 au = 1* $e \cdot a_o^{-3}$.

	Li-F	Na-F	K-F	Rb-F	Cs-F
	ωB97XD / aug-cc-pVTZ; (ECPs for K, Rb, Cs and I)				
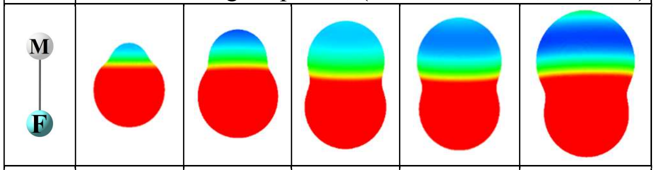					
x/au =<br>*y/au* =	+0.050<br>+0.340	+0.050<br>+0.230	+0.050<br>+0.140	+0.050<br>+0.105	+0.050<br>+0.080
	ωB97XD / def2-TZVPP; (ECPs for Rb, Cs and I)				
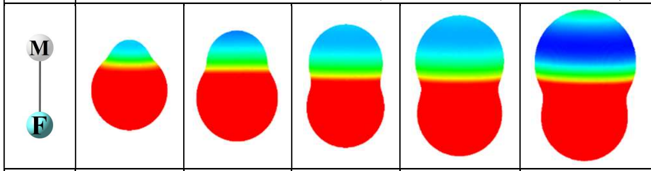					
x/au =<br>*y/au* =	+0.050<br>+0.340	+0.050<br>+0.230	+0.050<br>+0.140	+0.050<br>*+0.118	+0.050<br>+0.080
	ωB97XD / (All-electron)dyall-acv3z				
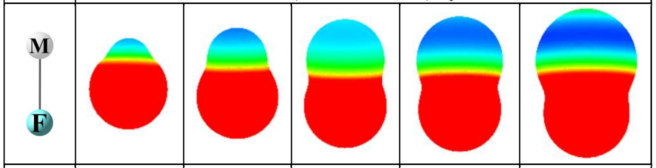					
x/au =<br>*y/au* =	+0.050<br>+0.340	+0.050<br>+0.230	+0.050<br>+0.140	+0.050<br>+0.105	+0.050<br>*+0.076
	ωB97XD / (All-electron) x2c-TZVPPall				
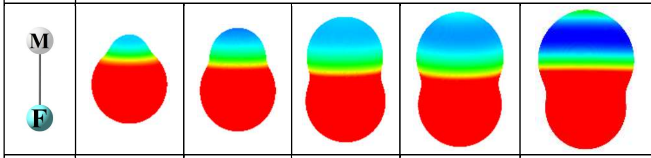					
x/au =<br>*y/au* =	+0.050<br>+0.340	+0.050<br>+0.230	+0.050<br>+0.140	+0.050<br>+0.105	+0.050<br>*+0.070
	ωB97XD / (All-electron) ANO-RCC-VTZP				
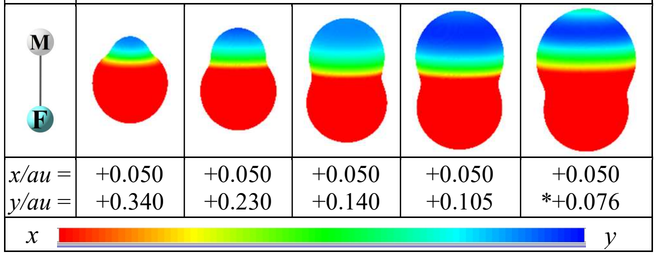					
x/au =<br>*y/au* =	+0.050<br>+0.340	+0.050<br>+0.230	+0.050<br>+0.140	+0.050<br>+0.105	+0.050<br>*+0.076
x					*y*

Figure S2: *Computed ESP maps (on the 0.001 au iso-surface) for MF molecules at five different levels of theory. For each MF molecule, the ESP x/y color ranges were selected to make the positive extrema on M as prominent as possible. *Slightly different 'y' values were used for some of the RbF and CsF cases (relative to the first image in those columns) in order to improve contrast for a given level of theory.*

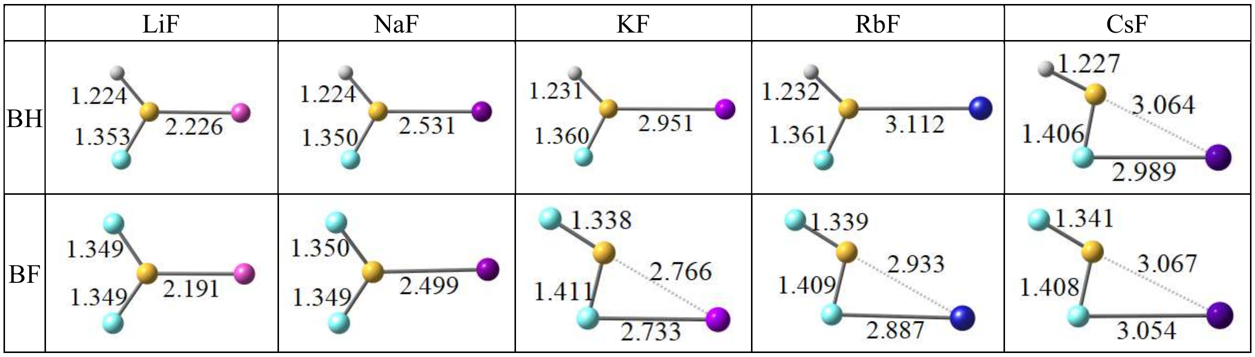

Figure S3: *Alternative structures to the sigma hole complexes identified in the main text. These structures were all optimized (starting with a triangular arrangement such as that which was located for CsF + BR above) and confirmed to be local minima at the same level of theory: ωB97XD/def2-TZVPP. Identities of atomic centers: H (grey), Li (yellow), F (light blue), and metal atoms (each identified in the first row) span pink to purple.*

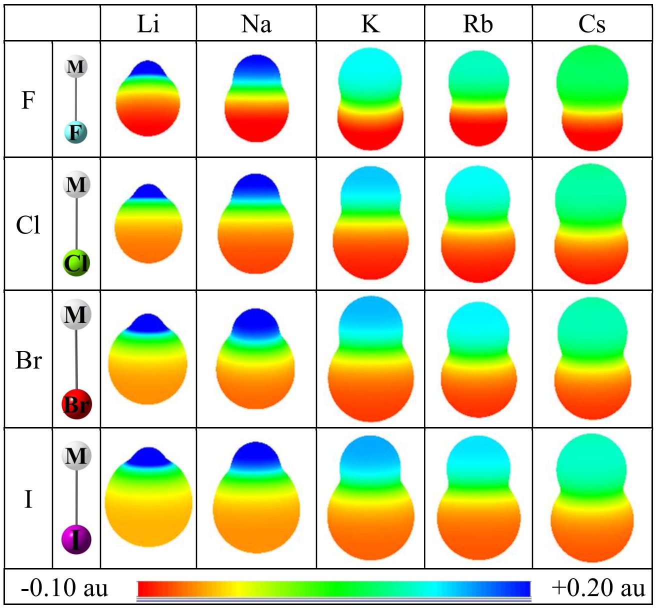

Figure S4: *Computed surface potentials (on the 0.001 au iso-surface) obtained at the MP2(full)/def2-TZVPP level for the group 1 metal halides.*

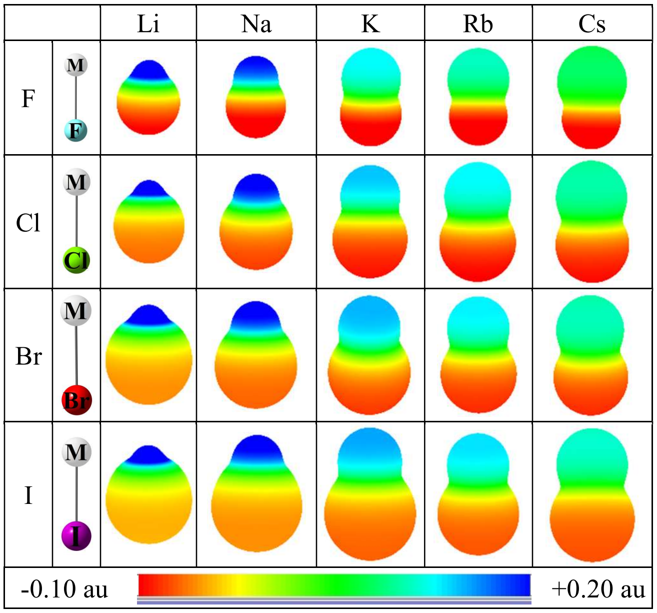

Figure S5: *Computed surface potentials (on the 0.001 au iso-surface) obtained at the CCSD(full)/def2-TZVPP level for the group 1 metal halides.*
